# Cardiovascular magnetic resonance in pulmonary hypertension

**DOI:** 10.1186/1532-429X-14-6

**Published:** 2012-01-18

**Authors:** William M Bradlow, J Simon R Gibbs, Raad H Mohiaddin

**Affiliations:** 1 Cardiovascular Magnetic Resonance Unit, Royal Brompton Hospital, London, UK; 2 Pulmonary Hypertension Service, Hammersmith Hospital, London, UK

**Keywords:** Right ventricle, pulmonary hypertension, pulmonary arterial hypertension

## Abstract

Pulmonary hypertension represents a group of conditions characterized by higher than normal pulmonary artery pressures. Despite improved treatments, outcomes in many instances remain poor. In recent years, there has been growing interest in the use of Cardiovascular Magnetic Resonance (CMR) in patients with pulmonary hypertension. This technique offers certain advantages over other imaging modalities since it is well suited to the assessment of the right ventricle and the proximal pulmonary arteries. Reflecting the relatively sparse evidence supporting its use, CMR is not routinely recommended for patients with pulmonary hypertension. However, it is particularly useful in patient with pulmonary arterial hypertension associated with congenital heart disease. Furthermore, it has proven informative in a number of ways; illustrating how right ventricular remodeling is favorably reversed by drug therapies and providing explicit confirmation of the importance of the right ventricle to clinical outcome. This review will discuss these aspects and practical considerations before speculating on future applications.

## Background

Pulmonary hypertension (PH) is the term given when mean pulmonary artery pressure (mPAP) is greater or equal to 25 mmHg [1]. It also describes a collection of clinical conditions in which pulmonary artery pressures are elevated.

At the most recent World Health Organisation conference (Dana Point, California) in 2008, PH was classified into five distinct disease groups (Table 1). Group 1 features patients with pulmonary arterial hypertension (PAH) and represents the most widely investigated subset of PH. It is thought to occur in 15-50 persons per million. Although newer treatments have led to improved survival, the disease continues to be associated with a poor prognosis [2].

**Table 1:** Classification of PH and Pointers to Each Subset

Dana Point Clinical Classification		Flags	Suggested Sequences
		
Group	Name		
1.	PAH	Shunts, aberrant pulmonary veins	Dedicated views, flow mapping and MRA
2.	Left heart disease	LV impairment, hypertrophy or wall motion abnormalities, dilated left atrium, valvular disease Pulmonary vein stenosis	LGE and valve lesion quantification Cine SSFP, flow and MRA
3.	Lung diseases and/or hypoxia	Emphysematous or fibrosed lung fields. However compared to high-resolution computed tomography, CMR images lungs poorly	
4.	CTEPH	Filling defects in the pulmonary arteries	MRA
5.	Unclear and/or multifactorial mechanisms	Lymphadenopathy Absent spleen	LGE

PAH; Pulmonary arterial hypertension, CTEPH; chronic thromboembolic pulmonary hypertension, MRA; magnetic resonance angiography, LGE; late gadolinium enhancement SSFP: steady state free procession.

For each patient, a methodical approach is necessary to describe the cause(s) of PH. Alongside right heart catheterisation, multimodality imaging plays a key part in this [3]. Echocardiography is the best screening test for PH whilst radioisotope ventilation-perfusion scanning remains the principal modality for the diagnosis of chronic thromboembolic pulmonary hypertension (CTEPH, Group 4). In patients with CTEPH being considered for pulmonary endarterectomy, computed tomography (CT) helps determine operative suitability. High resolution CT is used to characterize lung disease.

It is twenty five years since the use of cardiovascular magnetic resonance (CMR) was first reported in PH [4]. Due to its spatial resolution and freedom from acoustic windows, it has emerged as the gold standard for assessment of right ventricular (RV) structure and function [5,6]. This is particularly important in PH where the RV has long been thought to be important to survival [7]. Despite this, current guidelines rank other modalities more highly [1]. Though not surprising given CMR's limited availability and evidence base, experience with this technique is accumulating rapidly. It therefore seems unlikely that its position in future guidelines will remain unchanged.

The CMR examination is focused on the right heart and pulmonary arteries which undergo characteristic changes in response to elevated pulmonary pressures (Table 2). It is thought that RV hypertrophy initially predominates to compensate for pressure overload [1] before RV dysfunction and dilatation occur. The development of tricuspid regurgitation exacerbates volume overload. RV failure causes right atrial (RA) pressure [7] and size [8] to increase; both of which are related to outcome. Falling RV stroke volume and abnormal interventricular septal motion attenuate left ventricular (LV) filling and stroke volume. The proximal PAs become dilated. Histologically evident structural changes include media thickening and accumulation of mucopolysaccharide ground substance [9].

**Table 2:** Right Heart Changes in Pulmonary Hypertension

RV hypertrophy involving the papillary muscles, trabeculations and interventricular septum [72]. Asymmetric septal hypertrophy may be present [73-75]
Progressive RV dilatation until it becomes the dominant, apex-forming ventricle

Abnormal interventricular septal motion

Tricuspid regurgitation as a consequence of RV dilatation and stretching of the valve annulus

Interatrial septum becomes convex leftwards reflecting elevated RA pressures

Dilated RA

Plethoric vena cavae

Pericardial effusion

## Practical Considerations for CMR in PH

### Indications, Protocol and Interpretation of Findings

Patients may be referred with an established diagnosis of PH, with a diagnosis that requires clarification or with PH of unknown aetiology. Conversely, features of PH may be unexpectedly encountered in patients referred with left heart disease (where they should be reported due to its prognostic importance) or in patients with dilated RVs previously thought to have arrhythmogenic right ventricular dysplasia.

A minimum data set should allow RV size and function to be assessed by quantifying biventricular volumes, ejection fraction (EF) and mass. RV and LV stroke volume can be derived by flow mapping of the main pulmonary artery and aorta respectively. When reporting cardiac output and index, flow mapping of the aorta should be used [10] since it is smaller, has more coherent flow patterns and less translational movement than the main pulmonary artery. In instances where thromboembolic disease has not previously been excluded, an MR angiogram (MRA) of the pulmonary arteries should be undertaken. The examination is completed with imaging of late gadolinium enhancement (LGE). A suggested protocol is shown in Table 3.

**Table 3:** Suggested Imaging Protocol in PH

Sequence	Objective	Slice	
		
		Prescription	Parameters
**Localisers**	Identify the position of the heart	Sagittal, coronal and axial planes	

**Cines**	Define axes of both ventricles and the great arteries	HLA, VLA, SAX stack, LVOT, LVOT cross cut, RV VLA, MPA, MPA cross cut	Retrospectively gated, steady state free procession, slice thickness 7 mm, interslice gap 3 mm, FOV read 340 mm, FOV phase 75 mm

**Flow Measurements**	To determine stroke volume through main pulmonary artery and aorta	MPA/Ao	Retrospectively gated,2D segmented Spoiled Gradient Echo sequence. Slice thickness 10 mm, FOV read 350 mm, FOV phase 100 mm

**Magnetic Resonance Angiography**	To assess the pulmonary arterial tree	Ensure coverage of the lung vasculature	Non-ECG gated 3D Spoiled Gradient Echo sequence, slice thickness 1.30 mm, FOV read 400 mm, FOV phase 100 mm

**Late Gadolinium Enhancement**	To exclude areas of infarction and determine the degree of insertion region enhancement	Short axis stack and long axis acquisitions	2D segmented Spoiled Gradient Echo sequence with non-selective inversion pulse sequence. Slice thickness 8 mm, FOV read 340 mm, FOV phase 75 mm. LGE TI 260 mm initially then alter accordingly

HLA; horizontal long axis, VLA; vertical long axis, SAX; short axis, LVOT; left ventricular outflow tract, RV; right ventricular, MPA; main pulmonary artery, Ao; aorta, FOV; field of view, LGE; Late Gadolinium Enhancement, TI, inversion time.

It is not possible to consistently identify a cause by CMR alone. However, in selected cases a specific underlying disease may be suggested by the presence of one or more features (Table 1). It is important to integrate these findings with the examination, being prepared to deviate from this protocol to allow a more detailed assessment at the same sitting.

**Figure 1:**
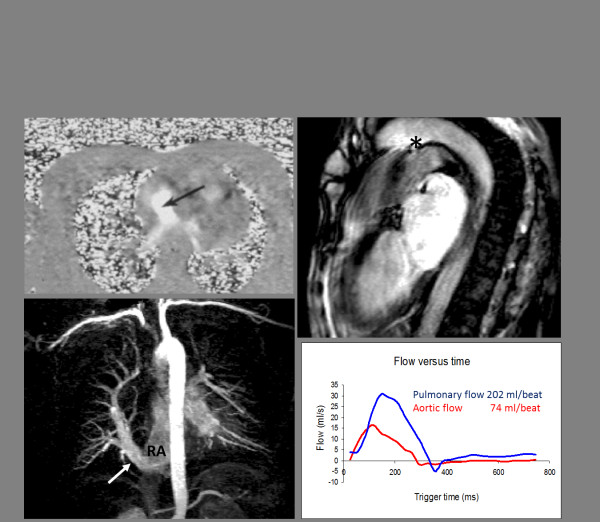
**Cardiovascular Magnetic Resonance in Group 1 PAH due to Congenital Heart Disease**. Top left, In plane flow mapping demonstrating flow between left and right atrium (arrow) through an atrial septal defect; Top right, a steady state free procession cine showing flow (asterisk) from descending aorta to pulmonary artery via a persistent ductus arteriosus; Bottom left, Magnetic Resonance Angiography of an aberrant pulmonary vein (arrow) draining into the right atrium (RA); Bottom right, flow mapping in this patient in the main pulmonary artery and aorta allowed a Qp:Qs of 2.7 to be derived.

**Figure 2:**
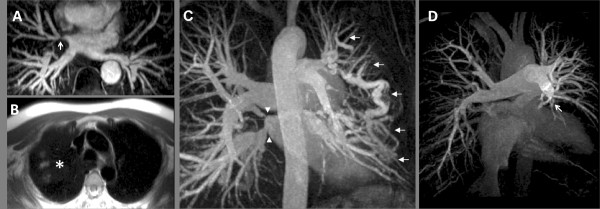
**Abnormalities of Pulmonary Vasculature in Pulmonary Hypertension.**; (A) White blood anatomy showing right upper pulmonary vein stenosis at the site of a prior ablation for atrial fibrillation (arrow). (B) Congestion and infarction in the right upper lobe on Half-Fourier Acquisition Single-Shot Turbo Spin-Echo images in the same patient (asterisk). (C) Magnetic Resonance Angiography from a separate patient with PH due to fibrosing mediastinitis; a varix is seen bypassing a stenosed left upper pulmonary vein (not shown) alongside stenoses of both right sided pulmonary veins (arrows). (D) Magnetic Resonance Angiography in patient with Chronic Thromboembolic Pulmonary Hypertension. The most striking feature is loss of the left descending pulmonary artery (arrow head).

In patients with features suggestive of shunt, dedicated anatomical imaging, cines, flow mapping and angiography should be undertaken. CMR is particularly helpful in the diagnosis of a sinus venosus defect and partial anomalous venous drainage [11] (Figure 1). If evidence of myocardial infarction or significant valvular heart disease exists, left heart causes (Group 2) become more likely. Diastolic dysfunction should be considered when LV hypertrophy and left atrial dilatation are found in the absence of other causes, especially in patients with diabetes and systemic hypertension. Pulmonary vein stenosis, either idiopathic or occurring after atrial fibrillation ablation therapy [12], can cause PH. The severity of the stenosis can be quantified by flow mapping and visualized with MRA (Figure 2).

Chronic thromboembolic disease can be identified using MRA (Figure 2). However, thrombosis arising in-situ can also be found in non-Group 4 patients, particularly in those with Eisenmenger syndrome [13]. More rarely, sarcomas can mimic pulmonary emboli. T1 weighted imaging following injection of gadolinium [14] may be helpful in differentiating the two. A combination of mediastinal lymphadenopathy and a non-coronary pattern of LGE is suggestive of cardiac sarcoid. An absent spleen is an important finding as it is associated with a higher risk of PH [15].

### Acquisition of Volumetric Data

**Figure 3:**
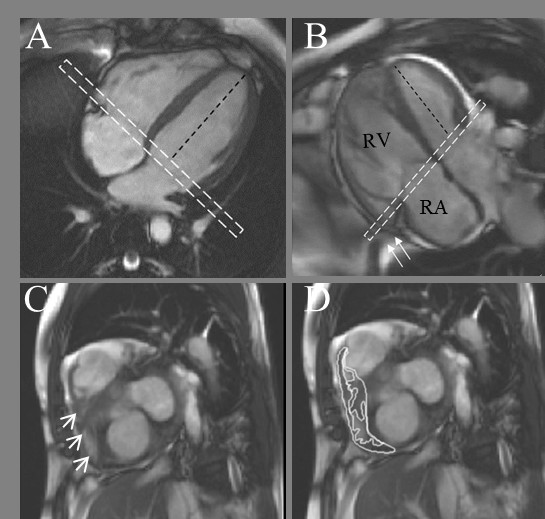
**Basal Slice Analysis**. In the normal right ventricle (A) the angle between the left ventricular long axis (black dashed line) and basal slice (dashed white box) is approximately 90°. In PH patients (B), assuming the basal slice is prescribed as per recommendations [16] the angle becomes more obtuse as the right ventricle dilates. The basal slice will now include right atrium (seen centrally) with a shoulder of right ventricle seen laterally (C, arrow heads). The impact this interpretation has on delineation is shown in D. Also in B note how the most basal part of the right ventricle 'hoods' beyond the TV plane (arrows) and is effectively ignored by the acquisition.

In routine practice, volumetric data is acquired by aligning the short-axis stack to the LV [16]. In PH, this creates several difficulties. Firstly the RV can dilate beyond the atrioventricular groove, when it appears as a 'shoulder' surrounding the right atrium in the basal short axis. In these cases, end-diastolic RV volume will be underestimated unless the short-axis starts within the atria (Figure 3). Secondly, in severe RV systolic impairment, atrioventricular excursion may not exceed slice thickness. In these instances, the basal slice may contain RV instead of RA at end-systole. To help discriminate RV from RA, atrioventricular excursion should be estimated by cross-referencing short-axis slices against each long axis acquisition [17,18]. Tracking an object's motion between slices and frames may also be useful.

It has been suggested that by orientating ventricular acquisition axially [19] or perpendicular to the long axis of the RV [20] these problems will be circumvented. Whilst these approaches improve interobserver reproducibility [19] and reduce differences between right and left ventricular volumes [20], they have not been adopted more widely. In addition, values obtained in this manner cannot be easily compared with normal values established using 'biventricular' analysis [21,22]. These factors argue in favor of the continued use of conventional analysis in patients with PH.

### Analysis of Right Ventricular Indices

There is no consensus as to how RV analysis should be performed. This has become an increasingly relevant issue as enhanced spatial resolution has allowed individual RV papillary muscles and trabeculae to be visualized. Acknowledging that these structures and the interventricular septum hypertrophy in PH, in our centre total RV mass includes the papillary muscles and trabeculae, as well as the 'RV' septum [23] when the interventricular septum is hypertrophied.

Manual analysis offers better control over delineation than semi-automated analysis, reflected in improved interobserver reproducibility [23]. However it is time consuming and requires end-systole to be predefined. This can be difficult in PH due to abnormal interventricular septal motion. One labor-intensive approach is to determine biventricular volumes in the frame exhibiting the most abnormal septal position, and the four preceding it, before choosing the smallest volume as end-systole for each ventricle. Alternatively, end-systole can be defined using valve opening and closure.

To simplify RV assessment, surrogate measurements have been developed. One candidate is analysis of transverse motion, which proved to be more strongly correlated with ejection fraction than tricuspid annular plane systolic excursion [24].

### Late Gadolinium Enhancement

**Figure 4:**
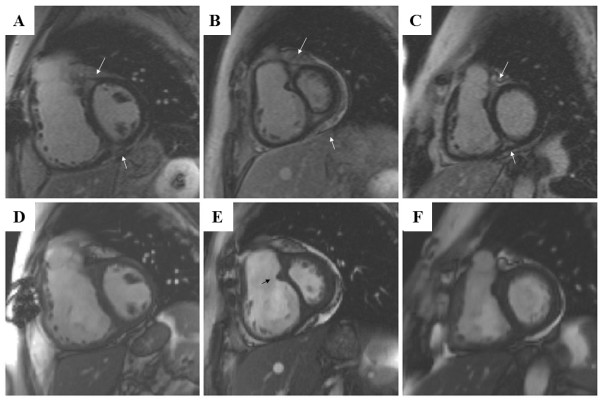
**The characteristic late gadolinium enhancement pattern of PH**. (A-C) insertion region enhancement (arrows) is triangular in shape with the base at the epicardial surface where both ventricles meet and its apex directed into the interventricular septum. Corresponding short axis cine slices (D-F). The septomarginal trabeculation is arrowed (E) - enhancement is often seen within this structure.

The finding of LGE limited to the insertion regions (Figure 4) has been repeatedly demonstrated [25-27] in PH since first described in 2005 by McCann and colleagues [28]. This has led to speculation that it may reflect pathological fibrosis [25] and hence be a source of ventricular arrhythmias [26]. Proving its histological basis has been difficult. In one of the very few pathology studies of the heart performed in PH, this area was not inspected [29]. In addition, its position is inaccessible to in-vivo biopsy. Pathological correlation in a patient who had died 6 weeks after CMR [30] showed myocardial disarray and plexiform fibrosis at the insertion regions where LGE occurred. These histological features are normally found in the insertion regions since they represent crossing points for left and right ventricular fibres with collagen in between [31]. Hence LGE may reflect pooling of gadolinium within an area of normal myocardium whose architecture has been accentuated by hypertrophy and mechanical stress.

## Non-Invasive Assessment of Haemodynamics

**Figure 5:**
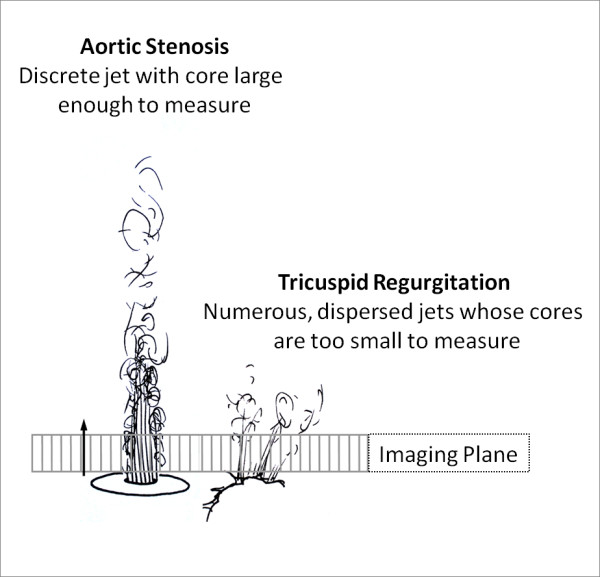
**Chasing Tricuspid Regurgitation**. Accurate jet velocity analysis depends on two factors; positioning of the imaging slice through the jet core and jet core characteristics. The latter is where measurement of tricuspid regurgitation with CMR falls down; compare the discrete stenotic lesion as might be seen in aortic stenosis (left) to the dispersed jets of tricuspid regurgitation (right) and how they relate to the imaging plane (represented by boxed line with direction of velocity measurement arrowed, modified with permission [32]). In functional tricuspid regurgitation, the jets arise at a number of points of failed coaptation and so are numerous and narrow - meaning their core is rarely large enough to be measured.

The ability to non-invasively estimate pressures within the right heart is a key objective since it would permit diagnosis and serial assessment without cardiac catheterization. Unfortunately, unlike echocardiography, it is not possible to generate an estimate of RV systolic pressure by measuring the velocity of the tricuspid regurgitant jet. This is because the tricuspid regurgitant jet is dispersed, so it is difficult to isolate the highest velocity in either through-plane or in plane acquisitions [32] (Figure 5).

Pulmonary pressures have been estimated by measuring the effects of PH on the heart. Initial results were encouraging for RV mass [33] but have been mixed subsequently [34]. Based on recent results, the degree of septal displacement may be a more promising measure [35].

CMR derived flow in the main pulmonary artery has also been used to gauge hemodynamics. For example, pulmonary pressures were shown to be inversely correlated with average blood velocity in the main PA [36]. In addition, total pulmonary resistance has been estimated by determining the percentage of regurgitant flow and cross-sectional area of the main pulmonary artery [37], or calculating the ratio of the maximal change in flow rate during ejection to the acceleration volume [38]. Finally, the use of four dimensional flow [39] has built on early work in two dimensional flow [40], to show that in patients with PH, a vortex can be detected in the primary flow direction whose duration correlates well with mPAP.

End-organ effects on the PAs themselves can also be assessed since their distensibility is reduced in PH [41]. It has been shown that a fractional change in the cross-sectional area of the main PA of less than 40% has a high sensitivity for detecting elevated mPAP [42]. Additionally, compliance has been calculated by combining velocity-encoded data and cross-sectional area change of the main PA and deriving pulse pressure through an iterative process [43]. From this, pulse wave velocity (PWV, a measure of vessel stiffness) was derived which had a reliability percentage of 87% for framing the actual mPAP.

**Figure 6:**
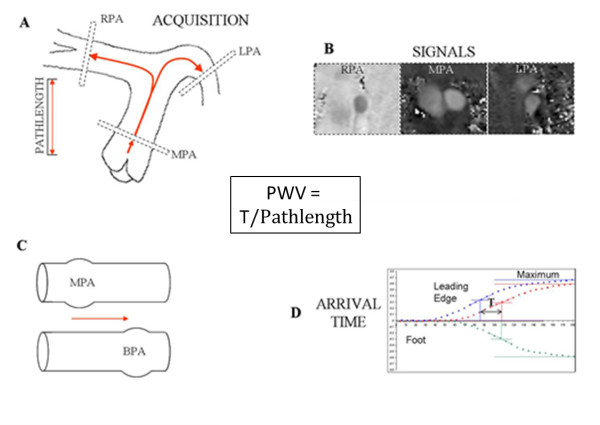
**Measuring Transit-Time PWV in the Pulmonary Arteries**. Data is acquired in main pulmonary artery, left and right pulmonary artery (A) and the path length between them measured accurately. Using CMR phase-contrast velocity maps (B), the flow pulse is tracked (C) and differences (T) in arrival time (D, in this healthy case defined as halfway between the foot and maximum values) are determined. PWV is then calculated as T/Pathlength. MPA; main pulmonary artery, RPA; right pulmonary artery, LPA; left pulmonary artery, BPA; branch pulmonary artery, PWV; Pulse wave velocity.

PWV can be calculated directly using the transit time technique by determining flow wave arrival time at two points in the proximal PAs using a high-temporal resolution flow mapping sequence (Figure 6), and dividing the difference by the distance between them [44]. This calculation does not depend on a prior knowledge of PA pressure and raises the possibility of entirely non-invasive assessment of PA stiffness.

The sheer number of indices emphasizes the lack of a single, robust non-invasive measure. Indeed in one independent validation [34], none of the tested parameters accurately predicted mPAP. For CMR to replace cardiac catheterization, in addition to right heart pressures, left atrial pressure also needs to be estimated. Early work has shown this might be possible through the measurement of transmitral flow and myocardial tissue velocity (i.e. akin to tissue Doppler imaging in echocardiography) [45].

## Determining Treatment Effect and Prognosis

CMR is well suited to longitudinal follow up as it is non-invasive and non-ionizing. Several studies have reported the positive effects medical [46-49] and surgical [50-54] therapies have on RV structure and function. CMR has also been used to determine which patients with idiopathic PAH might benefit from long-term calcium channel blockade by assessing main PA distensibility [55].

This has driven interest in the use of CMR to measure efficacy of new therapies. To date, two proof of concept trials [48,56] have used CMR-derived RV mass as an end-point. This particular surrogate remains unvalidated and may not be the best end-point since it is not known whether a reduction in RV mass is beneficial or harmful to patients.

Using CMR as a trial end-point remains an attractive option since it avoids the limitations associated with the most widely use surrogate in PAH trials; the six minute walk test distance [57]. In addition, fewer patients are required due to its high interstudy repeatability [23].

The RV has been implicated in clinical outcomes from the earliest right heart catheterization studies which showed parameters which could be related to this ventricle (cardiac output, RA pressure and mixed-venous oxygen saturations) were relevant to prognosis [7,58]. However it has only been with maturation of single-centre experience (the VU University Medical Center in Amsterdam, The Netherlands) that this assumption has been proven with CMR.

**Figure 7:**
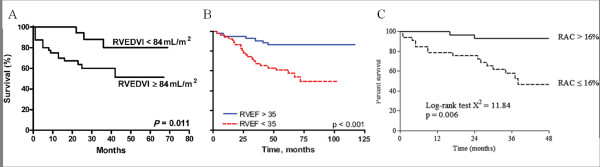
**The RV-PA unit and Survival in PAH.** Work from the VU University Medical Center (Amsterdam) group demonstrating the importance of right ventricular dilatation [59] (A), ejection fraction[60] (B), and pulmonary artery stiffness [61](C) to prognosis. RVEDVI; indexed right ventricular end-diastolic volume, RVEF; right ventricular ejection fraction, RAC; relative area change.

The first outcome study to be reported with CMR [59] demonstrated the importance of indexed biventricular dimensions; a relationship that had not been hitherto revealed by echocardiographic studies. Indexed RV end-diastolic volume was prognostic both at baseline (Figure 7) and at 1 year as were indexed stroke volume and LV end-diastolic volume. These data have recently been extended to include RV ejection fraction [60]. CMR parameters of PA stiffness are also relevant to prognosis [61] (Figure 7). This emphasizes the point that as the PA become stiffer their ability to buffer pulsatile blood is lost. The RV must compensate for this by generating additional energy to propel blood downstream.

## Providing Pathophysiological Insights

CMR has offered a number of wide-ranging insights into the pathogenesis of PH through diverse applications (Table 4). By measuring PA distensibility, it has been shown that in patients with normal pulmonary pressures at rest but detectable PH with exercise, the PAs are stiffer than in normal subjects [42]. The authors speculated that this increased stiffness may itself play a role in accelerating pulmonary vascular remodeling. CMR has demonstrated that left ventricular mass is lower than normal in patients with CTEPH before pulmonary endarterectomy but normalizes afterwards [62]. This is thought to be due to myocardial apoptosis of the chronically underfilled LV. Using tagging, it has been observed that the RV in PH contracts more slowly than the LV creating interventricular dyssnchrony and subsequent interventricular septal bowing [63].

**Table 4:** New Insights Provided by CMR

INSIGHT	CMR METHOD	REF#
**Early Changes**		

PA stiffens before pulmonary artery pressure increase at rest	Distensibility of pulmonary arteries	[42]

**Ventricular Remodeling and Dysfunction**		

LV mass is lower in patients with chronic thromboembolic disease but normalizes post-pulmonary endarterectomy	LV mass	[62]

Interventricular dyssnchrony and septal bowing in PAH is due to slower contraction for the RV than LV	Tagging	[63]

**Cardiac Ischemia**		

Both ventricles display attenuated vasoreactivity proportional to mPAP	Adenosine Stress Perfusion	[64]

**RV Metabolism**		

Bosentan improves RV energetics	31P-NMR spectroscopy	[65]

**Ventricular-arterial Decoupling in PH**		

Disconnect occurs since increases in arterial load are far greater than those in contractility	Volumes combined with invasively derived pressure loops/pressures	[66,67]

**Changes with Exercise**		

Stroke volume in PAH fails to augment - increases in cardiac output are mediated by heart rate alone	Bicycle exercise	[68]

**Inaccuracy of Catheter-Laboratory Measurements**		

Pulmonary vascular resistance derived from the Fick method is inaccurate in conditions of vasodilatation	Flow combined with invasively derived pressure	[69]

CMR; cardiovascular resonance imaging, PH; pulmonary hypertension, LV; left ventricle, RV; right ventricle, mPAP; mean pulmonary artery pressure, PAH; pulmonary arterial hypertension

During CMR perfusion with adenosine stress in patients with PH, biventricular vasoreactivity has been found to be diminished. The degree to which this occurs in both ventricles could be predicted from mPAP [64]. Despite its potential, nuclear magnetic resonance (NMR) spectroscopy remains relatively unexplored in PH. An isolated case report using 31P-NMR spectroscopy offers a unique insight into the failing RV by showing how RV energetics are disturbed in PAH but improve with bosentan treatment [65].

Interventional CMR allows RV pressure-volume loops to be created from which three key measures are extracted: 1) Systolic function (Emax; end-systolic pressure divided by end-systolic volume, 2) Afterload (Ea; end-systolic pressure divided by stroke volume) and 3) Ventricular-arterial coupling (Emax/Ea). In patients with PAH, Kuehne and colleagues [66] found that while systolic function was increased, the increase in arterial elastance was relatively greater leading to ventricular-arterial decoupling. These findings have been echoed more recently in a larger cohort of patients with PH (albeit using different definitions of Emax and Ea) [67].

Exercise CMR, performed on a recumbent bicycle within the scanner, has been used to demonstrate that exercise-induced increases in cardiac output in patients with idiopathic PAH are due to heart rate changes alone, since stroke volume fails to augment [68]. CMR has also detailed inaccuracies with the Fick method for determining cardiac output in conditions of high pulmonary blood flow or increased oxygen concentration [69].

## Future Directions

CMR is unlikely to be more widely integrated into a general imaging pathway in PH until its incremental value is proven in routine clinical practice. To determine whether this is the case, clinical trials of CMR imaging should be undertaken comparing standard care in PH with that informed by CMR results. It is also important that analysis become standardized and facilitated by improved software.

It has also been suggested that CMR be used to 'screen' potential therapies before they are selected for definitive, long-term studies [70]. Before doing so, CMR parameters need to be validated [71]. One aspect of this involves determining which of the available measures best reflects prognosis. This should include an exploration of load-independent end-points, such as ventricular tagging.

Like most research in PH, CMR-based work has been mainly applied to Group 1 (PAH). It remains unclear whether the RV in each group behaves differently in the face of similar pulmonary pressures. Through contemporaneous assessment of the RV-PA functional unit, CMR might be able to contribute to an answer, and if this assertion is correct, explain why.

Work should continue to define the relevance of flow wave morphology and pulmonary artery stiffness with focus extended to non-invasively determining left atrial pressure. The opportunity to validate this work is offered by the interventional CMR suite. Finally, small animal CMR is being explored although it should be acknowledged that animal models of PAH are unreliable in predicting human responses.

## Conclusions

CMR permits an accurate assessment of the right ventricle and pulmonary artery flow. In current clinical practice, it is an especially useful tool in patients with a diagnosis of (or suspected of having) PAH associated with congenital heart disease. However it also allows discrimination of other features which might point to an alternative etiology. It is a flexible research tool which has opened new avenues for understanding treatment effects, outcomes and pathogenesis. It appears inevitable that its place in the management of PH will evolve as evidence supporting its use accumulates.

## List of Abbreviations

CMR: Cardiovascular Magnetic Resonance; CTEPH: chronic thromboembolic pulmonary hypertension; EF: ejection fraction; HASTE: Half-Fourier Acquisition Single-Shot Turbo Spin-Echo; LGE: Late Gadolinium Enhancement; LV: left ventricle; PAH: pulmonary arterial hypertension; mPAP: mean pulmonary arterial pressure; MRA: magnetic resonance angiography; PA: pulmonary artery; PH: pulmonary hypertension; PVR: pulmonary vascular resistance; PWV: Pulse wave velocity; RA: right atrium; RV: right ventricle; SSFP: steady state free procession.

## Competing interests

The authors declare that they have no competing interests.

## Authors' contributions

All authors participated in literature review, manuscript preparation and final approval of the submitted manuscript.
